# Transcriptomic analysis of the trade-off between endurance and burst-performance in the frog *Xenopus allofraseri*

**DOI:** 10.1186/s12864-021-07517-1

**Published:** 2021-03-23

**Authors:** Valérie Ducret, Adam J. Richards, Mathieu Videlier, Thibault Scalvenzi, Karen A. Moore, Konrad Paszkiewicz, Camille Bonneaud, Nicolas Pollet, Anthony Herrel

**Affiliations:** 1 UMR 7179 MECADEV, C.N.R.S/M.N.H.N., Département Adaptations du Vivant, 55 Rue Buffon, 75005 Paris, France; 2 Station d’Ecologie Expérimentale du CNRS, USR 2936, 09200 Moulis, France; 3 Functional Ecology Lab, Department of Biology, University of Ottawa, 30 Marie Curie, Ottawa, ON K1N 6N5 Canada; 4 Evolution, Génomes, Comportement & Ecologie, Université Paris-Saclay, CNRS, IRD, 91198 Gif-sur-Yvette, France; 5 Exeter Sequencing Service, College of Life and Environmental Sciences, University of Exeter, Exeter, EX4 4QD UK; 6 Centre for Ecology & Conservation, College of Life and Environmental Sciences, University of Exeter, Penryn, Cornwall, UK; 7 Evolutionary Morphology of Vertebrates, Ghent University, B-9000 Ghent, Belgium

**Keywords:** Anura, Limb, Muscle, Myosin, RNA-sequencing, Stamina

## Abstract

**Background:**

Variation in locomotor capacity among animals often reflects adaptations to different environments. Despite evidence that physical performance is heritable, the molecular basis of locomotor performance and performance trade-offs remains poorly understood. In this study we identify the genes, signaling pathways, and regulatory processes possibly responsible for the trade-off between burst performance and endurance observed in *Xenopus allofraseri*, using a transcriptomic approach.

**Results:**

We obtained a total of about 121 million paired-end reads from Illumina RNA sequencing and analyzed 218,541 transcripts obtained from a de novo assembly. We identified 109 transcripts with a significant differential expression between endurant and burst performant individuals (FDR ≤ 0.05 and logFC ≥2), and blast searches resulted in 103 protein-coding genes. We found major differences between endurant and burst-performant individuals in the expression of genes involved in the polymerization and ATPase activity of actin filaments, cellular trafficking, proteoglycans and extracellular proteins secreted, lipid metabolism, mitochondrial activity and regulators of signaling cascades. Remarkably, we revealed transcript isoforms of key genes with functions in metabolism, apoptosis, nuclear export and as a transcriptional corepressor, expressed in either burst-performant or endurant individuals. Lastly, we find two up-regulated transcripts in burst-performant individuals that correspond to the expression of myosin-binding protein C fast-type (*mybpc2*). This suggests the presence of *mybpc2* homoeologs and may have been favored by selection to permit fast and powerful locomotion.

**Conclusion:**

These results suggest that the differential expression of genes belonging to the pathways of calcium signaling, endoplasmic reticulum stress responses and striated muscle contraction, in addition to the use of alternative splicing and effectors of cellular activity underlie locomotor performance trade-offs. Ultimately, our transcriptomic analysis offers new perspectives for future analyses of the role of single nucleotide variants, homoeology and alternative splicing in the evolution of locomotor performance trade-offs.

**Supplementary Information:**

The online version contains supplementary material available at 10.1186/s12864-021-07517-1.

## Background

Locomotor performance has a strong impact on the survival and reproduction of many organisms [1–3]. Burst performance is often most relevant in the context of prey capture and predator escape, whereas endurance is relevant in the context of territory defense, dispersal, or migration. Yet, the evolution of locomotor performance can be constrained if performance traits are involved in traded-offs, as often observed between burst performance and endurance capacity in vertebrates [4–9]. Conflicting demands on muscles to express either fast-twitch glycolytic fibers that facilitate burst performance or slow-twitch oxidative muscle fibers that enhance stamina may explain in part this performance trade-off [10–13]. Although the physiological basis of this performance trade-off has been documented, how it is governed at the gene expression level remains poorly understood. Uncovering the molecular basis and biological pathways underlying performance trade-offs is therefore essential for understanding the adaptive evolution of these traits.

Because locomotor performance is heritable [14–16], efforts have been made to explain differences in physical performance by variation in coding DNA in humans [17–19], racing pigeons [20], mice [21], horses [22] and dogs [23]. While those studies highlighted a remarkable number of genetic variants associated with variation in physical performance, they provide little insight into the potential processes underlying performance trade-offs. Altogether, the myriad of genetic variants with little phenotypic effects has led to the consensus that physical performance is a polygenic trait that is governed by features such as transcriptional regulation. Recently, microRNAs have been found to regulate the expression of target genes in skeletal muscle [24, 25], as well as target genes involved in muscle cell proliferation, differentiation, motility and regeneration [26]. In humans, a transcriptional map established after endurance exercise training highlighted an important regulation of gene expression to increase aerobic capacity [27]. Although a few transcriptomic analyses have been performed in the context of physical performance [20, 27, 28], none have tried to understand the factors underlying performance trade-offs.

In this study, we analyzed the transcriptomes of eight adult *Xenopus allofraseri* males from a single population that show a marked trade-off between endurance and burst-performance capacity. We performed a RNA-seq analysis of genes expressed in limb muscle that allowed us to highlight the genes, signaling pathways, and regulatory processes such as alternative splicing likely underlying this locomotor performance trade-off.

## Results and discussion

### Raw sequencing data, de novo assembly and quality control

We obtained a total of about 121 million paired-end reads using Illumina RNA sequencing. After trimming and quality filtering, biological replicates produced between 5.2 and 28 million paired-end reads (Table 1). The number of reads in each group was well balanced with 5.5 million in the endurant group and 6.6 million in the burst-performant group. The BUSCO analysis resulted in 65.4% gene identification (54.9% completeness and 10.5% of fragmented genes), which is relatively good as only one muscle tissue was sampled. Next, we evaluated the Trinity de novo assemblies by mapping the trimmed reads. We obtained an overall alignment rate of > 97% percent identity and > 89% of reads aligned as proper pairs (Table 1). The de novo assembly consisted of 218,541 transcripts and 163,981 ‘genes’ with an E90N50 value (i.e. the N50 for transcripts that represent 90% of the total normalized expression data) of 1462 pb. These different metrics testify that our transcriptome assemblies were of good quality.

**Table 1:** Summary of quality scores for the sequencing of the eight males *Xenopus allofraseri* (named sample A to H)

Sample	Paired-end reads	Total singleton reads	> = Q30 (%)	Mean quality score
A	8,961,655	17,923,310	94.02	36.42
B	16,672,325	33,344,650	93.97	36.41
C	28,509,462	57,018,924	93.71	36.31
D	14,145,979	28,291,958	94.07	36.44
E	20,664,890	41,329,780	94.19	36.49
F	15,697,497	31,394,994	94.06	36.44
G	11,647,327	23,294,654	94.37	36.54
H	5,288,288	10,576,576	94.00	36.41

Q30: Phred quality scores when probability of incorrect base call is 1 in 1000

### Physical performance

**Fig. 1:**
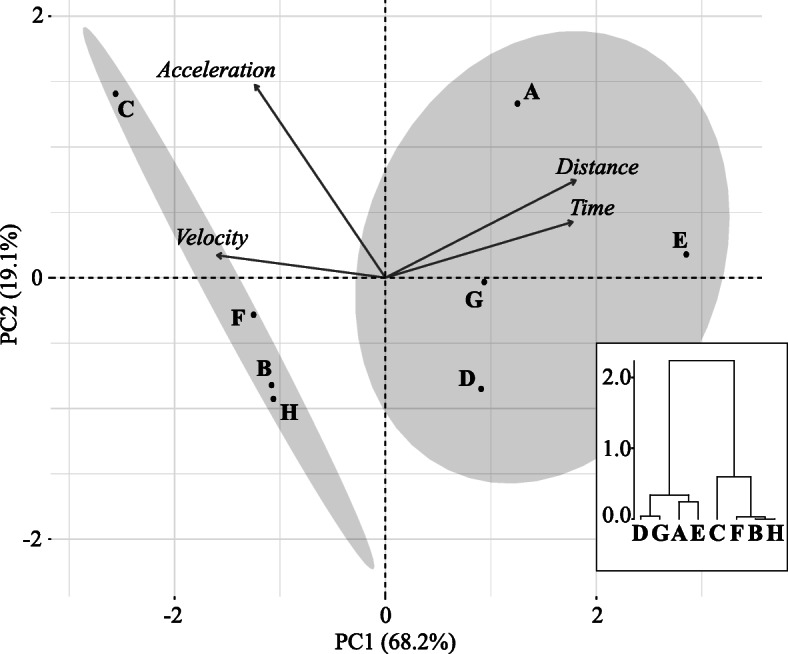
Principal Component Analysis (PCA) and agglomerative hierarchical clustering of the four locomotor performance traits in eight males *Xenopus allofraseri* (named sample A to H): distance (total distance jumped until exhaustion), time (maximum time spent moving until exhaustion), acceleration (maximal instantaneous acceleration during an escape locomotor burst), velocity (maximal instantaneous speed during an escape locomotor burst)

Transcript levels were quantified with respect to endurant and burst performant classifications after measuring four physical performance traits: maximum distance jumped before exhaustion (m), maximum time jumped before exhaustion (s), maximum burst velocity (m.s^− 1^), and maximum burst acceleration (m.s^− 2^) (Table 2). The principal component analysis (PCA) followed by the agglomerative hierarchical clustering allowed to clearly segregate individuals into the two groups (burst performant vs. endurant individuals; Fig. 1) confirming the existence of a locomotor trade-off in this species. Maximum distance, maximum time and maximum velocity contributed mainly to the first axis of the PCA (respectively 92.1, 90.5 and 81.3%), whereas maximum acceleration contributed to the second axis (75.3%).

**Table 2:** Individual measures of locomotor performance of the eight males *Xenopus allofraseri* (named sample A to H)

Sample	Category	Velocity (m.s^− 1^)	Acceleration (m.s^− 2^)	Time (s)	Distance (m)
A	Endurant	1.17	54.41	71	1.190
B	Burst-performant	1.67	47.75	46	0.530
C	Burst-performant	1.87	61.83	36	0.590
D	Endurant	1.10	45.17	55	0.840
E	Endurant	1.20	43.80	96	1.310
F	Burst-performant	1.87	49.69	54	0.575
G	Endurant	1.56	46.05	73	1.040
H	Burst-performant	1.44	48.42	32	0.560

### Phylogenetic analysis

**Fig. 2:**
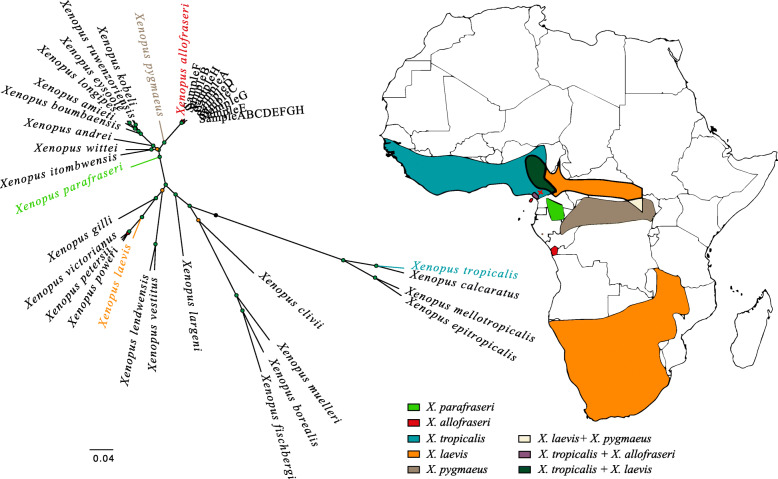
Geographic range of some *Xenopus* species in Africa and maximum-likelihood phylogenetic tree of the eight studied *Xenopus* males captured in Cameroon in 2009 (represented by a red cross). Geographic ranges were downloaded from the IUCN 2020 red list [29] and the map was created with QGIS v.3.14 (https://www.qgis.org/). The unrooted tree shows the phylogeny built with PhyML [30] based on mitogenomes assembled de novo (Sample A to H correspond to the reconstructed mitochondrial sequence based on each individual data whereas Sample ABCDEFGH corresponds to the reconstructed mitochondrial sequence from all individual data combined) and from mitogenomes of other *Xenopus* species previously published (corresponding GenBank accession numbers are presented in Table S2). The phylogenetic tree was designed using Figtree v.1.4.4 (http://tree.bio.ed.ac.uk/software/figtree/). The branch lengths are proportional to the number of substitutions per site with the scale indicated under the tree. The Shimoidara-Hasegawa (SH)-like branch support test is represented by node colors (*p-value* > 0.95 in green, *p-value* > 0.80 in orange, *p-value* < 0.80 in red)

Phylogenetic analysis of the mitogenomes indicated that mitochondrial DNA from the eight *Xenopus* males (Sample A to H, Fig. 2) are closely related and correspond to specimens of the species *Xenopus allofraseri*. These mitochondrial sequences are sister to those of *Xenopus pygmaeus* and markedly diverge from other *Xenopus* species such as *Xenopus laevis* and *Xenopus tropicalis*. Noticeably, the eight *Xenopus allofraseri* males were captured in a geographic range that was not previously reported for this species [29].

### Differentially expressed transcripts

**Fig. 3:**
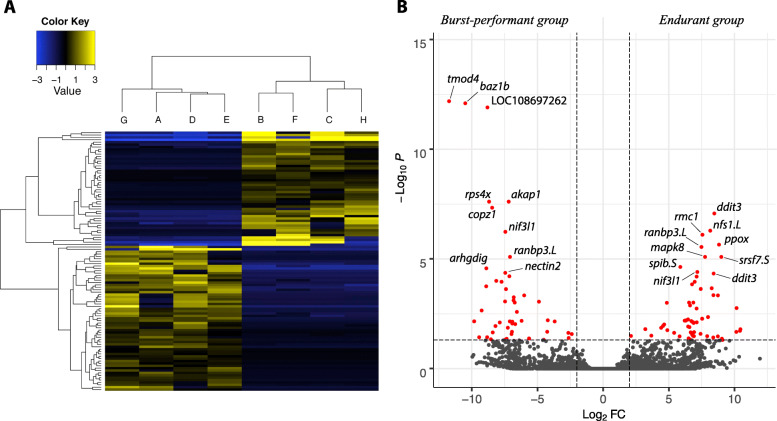
**a** Heatmap representation of the regularized log-transformed counts for the de novo assembly. All transcripts (*n* = 109) shown had significance levels with (FDR) ≤ 0.05. The expression values are plotted in log2 space and mean-centered, and show up- and down-regulated expression as yellow and blue, respectively. **b** Volcano plot of all de novo transcripts and the red data points corresponding to the significantly differentially expressed transcripts. Gene symbol of the top 10 most differentially expressed transcripts in endurant and in burst-performant groups are plotted

**Fig. 4:**
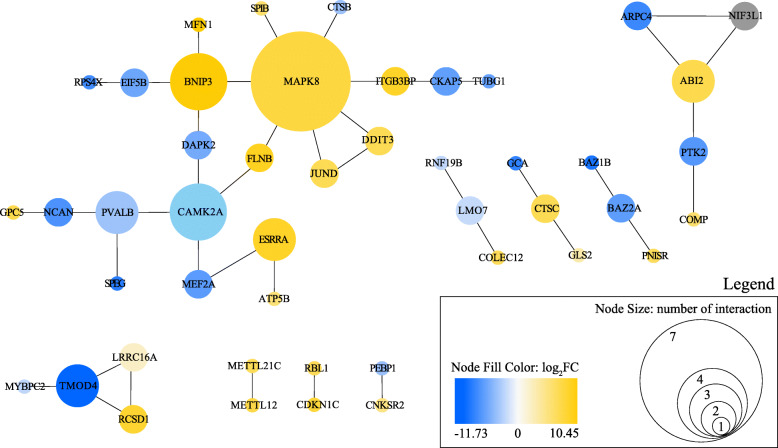
Gene interaction networks that contain 46/109 differentially expressed transcripts between endurant and burst-performant individuals. Differentially expressed transcripts were analyzed using STRING [31] using gene symbols of human orthologous genes for analysis (see the supplementary table to find corresponding *X. allofraseri* annotated transcripts), and visual inspection was finalized using Cytoscape [32]. The node color is based on the log_2_FC of expression data, with negative (blue) and positive (yellow) values representing up-regulated transcript expression in endurant and burst-performant individuals, respectively (grey color correspond to gene with transcript isoforms expressed in both groups). Node size represents the number of interactions with other protein-coding genes and allows to rapidly visualize central genes

We identified 109 transcripts with a significant differential expression between endurant and burst performant individuals (Fig. 3). Six of those transcripts yielded no similarities to either the Uniprot or the NCBI databases. The blast searches resulted in 103 protein-coding genes (Table S1) matching either *Xenopus laevis* (*n* = 94) or *Xenopus tropicalis* (*n* = 9) proteins. Due to alternative splicing, some transcripts blasted to the same gene, therefore we identified 90 unique protein-coding genes. Using the human STRING database, we generated nine networks involving 46 differentially expressed protein-coding genes (Fig. 4).

We highlighted differentially expressed protein-coding genes involved in the structural organization and functioning of muscle cells, such as actin cytoskeleton and microtubule composition, conformation, mitochondrial activity, and cellular trafficking. Yet, it appears that many of those transcripts have regulatory properties or are effectors of downstream signaling cascades, starting from stimuli in the extracellular matrix and involving cell surface or transmembrane proteins. Consequently, endurant and burst-performant individuals differ in the functional pathways that are initiated by those up-stream effectors.

#### Transmembrane proteins and focal adhesions

Focal adhesion are macromolecular assemblies that play key roles in linking the extracellular matrix to the cytoskeleton [33] and act as important signal transducer [34]. In loading muscle, previous study highlighted the role of focal adhesion kinase (FAK, PTK2 gene) to act as a mediator, and transmit a stress and strain signal by integrins (transmembrane receptors) that activate multiple anti-apoptotic, cell growth pathways [35] and increase muscle mass [36]. Whereas FAK lead to slow twitch muscle generation and to an up-regulation of genes involved in mitochondrial metabolism [37], FAK-related non-kinase (FRNK) - a protein transcribed from the FAT portion of the FAK gene - acts to inhibit FAK in many cell types, including skeletal muscle [38]. In our study, we find *ptk2* and other protein-coding genes (*gca, lmo7*) involved in focal adhesion and the signal transduction cascade through the activation of Rho-GTPases (e.g. RhoG, rac1, cdc42) to be up-regulated in burst-performant individuals. Also, kinectin 1 (*ktn1)*, a receptor for kinesin that accumulates in integrin-based adhesion complexes, is up-regulated in endurant individuals, whereas *mef2a,* a DNA-binding transcription factor of *ktn1,* is up-regulated in burst-performant individuals. Remarkably, kinectin interacts with RhoG to activate rac1 and cdc42 through a microtubule-dependent pathway [39]. Indeed, kinesins are major microtubule motor proteins that have different functional properties depending on the ‘cargo’ (i.e. vesicle) they transport. We found several genes involved in microtubule composition and elongation, such as *tubg1* and *ckap5,* to be up-regulated in burst-performant individuals. Those genes interact with a centromere protein R-like (an ortholog of *ITGB3BP*) that is up-regulated in endurant individuals (Fig. 4).

Furthermore, we found a differential expression of several protein-coding genes related to cellular trafficking and the Golgi apparatus. This central organelle system of the secretory pathway biosynthesizes proteoglycans [40]. It is also an important center for the formation of microtubules for its own functioning, also called ‘MTOC’ [41]. We found RhoGDI-3 (*arhgdig*) to be up-regulated in burst-performant individuals and it targets RhoG from the Golgi apparatus to be activated locally [42]. Interestingly, *arafgap1,* which codes for a GTPase-activating protein involved in membrane trafficking and vesicle transport from the Golgi complex, is up-regulated in endurant individuals. Yet, *arafgap1* interacts with *copz1* (Fig. 4), which codes for a coatomer (i.e., a protein complex that associates with Golgi coated vesicles and mediate transport from the endoplasmic reticulum). This protein-coding gene is up-regulated in burst-performant individuals, as well as *rab12* and *grasp,* which both play a role in intracellular trafficking. We emit the hypothesis that endurant and burst-performant individuals differ in a range of downstream effectors, transcription regulators, molecules involved in cellular trafficking and microtubule activity in order to biosynthesize distinct extracellular matrix molecules and cell surface proteins, such as proteoglycans in the Golgi apparatus.

#### Extracellular matrix and proteoglycans

The extracellular matrix (ECM) is a primary macrostructure composed of several molecules such as collagen, hyaluronan, proteoglycans and glycoproteins that assemble into an organized meshwork [31, 43]. Proteoglycans for instance have diverse and essential roles in matrix remodeling and can act as receptors or co-receptors to affect signaling pathways but also to initiate and modulate signal transduction cascades independently of other receptors [44, 45]. In this study, we highlighted the up-regulation of genes coding members of two large groups of proteoglycans: neurocan (*ncan*), a chondroitin sulfate proteoglycan that is up-regulated in burst-performant individuals, and glypican (*gpc5*), a heparan sulfate proteoglycan that is up-regulated in endurant individuals. In addition, we found an up-regulation of a cartilage oligomeric matrix protein-like (*comp)* in endurant individuals that has the molecular functions to bind calcium, heparin or proteoglycans. Therefore, it is plausible that endurant and burst-performant individuals differ in the proteoglycans and other extracellular proteins synthesized because their diversity and properties make them advantageous for powerful bursts of speed or long-duration exercise.

Chondroitin sulfates that partly compose aggrecan are able to absorb shocks by binding and releasing water content during compression in cartilaginous tissues, tendons, or ligaments [46, 47] which can protect against injury during short and powerful physical performance. In addition, it has been shown that glypican-1 is able to enhance growth factor activity and is therefore used in therapeutic treatment to create new vasculature and restore blood flow in ischemic tissues [48]. Therefore, there could be a link between *gpc5* and the positive relationship between endurance training and capillary densities [49], which may be beneficial for transporting oxygen to muscle [50]. Interestingly, we also found *ppox*, which codes for an essential component of hemoglobin and myoglobin, and *spib*, a hematopoietic transcription factor, to be up-regulated in endurant individuals. The coupling of increased blood oxygenation and muscle microvasculature is expected to render the aerobic pathway used during prolonged exercise more efficient. Finally, the study of Mao and colleagues [51] suggests that *spib* could be phosphorylated and activated by mitogen-activated protein kinase 8 (*mapk8)*, which is also up-regulated in endurant individuals, and is part of a vast network comprising numerous genes involved in lipid metabolism, mitochondrial activity, and stress responses.

#### Lipid metabolism, mitochondrial activity and stress response

Almost all differentially expressed transcripts related to lipid metabolism, energy production, mitochondrial activity (*mfn1, esrra, atp5b, dgat2, gls2, nfs1)* are up-regulated in endurant individuals compared to burst-performant individuals. Yet, one protein-coding gene, a A-kinase anchor protein 1 (*akap1*), has 2 transcript isoforms, one being up-regulated in burst-performant and one in endurant individuals. Those splice variants are proteins found in the mitochondria transmembrane, but at different position (position 7–26 and 42–61 in burst-performant and endurant individuals, respectively). The A-kinase anchor protein 1 binds to different regulatory subunits of protein kinase A (PKA) that has regulatory properties in lipid, sugar, and glycogen metabolism. Interestingly, we found an up-regulation of *fsd2* in burst-performant individuals, which is an important paralog of *CMYA5* that mediates subcellular compartmentation of protein kinase A and may attenuate the ability of calcineurin to induce a slow-fiber gene program in muscle [52]. Thus, we suggest that alternative splicing of *akap1,* in association with other mitochondrial or cytoplasmic genes, is a mechanism enabling the shift between different types of metabolism in endurant and burst-performant individuals.

Furthermore, our results are consistent with the fact that endurant individuals rely preferentially on lipid metabolism, because oxidative phosphorylation of fatty acids in muscle mitochondria produces a high yield of ATP, necessary for prolonged contraction of muscle fibers [53, 54]. On the contrary, individuals excelling at burst performance may rely mostly on anaerobic glycolysis in the cytosol (fast rate but low yield of ATP) [55]. In this context, we found diacylglycerol acyltransferase 2 (*dgat2*) to be up-regulated in endurant individuals. This endoplasmic reticulum enzyme catalyzes the final step in triglyceride synthesis and is part of the glycerolipid metabolism [56]. In addition, we found an up-regulation of *atp5b,* a mitochondrial ATP synthase subunit, by the estrogen-related receptor α (ERRα, coded by *esrra*) that regulates the transcription of metabolic genes and has a role in oxidative metabolism (Fig. 4) [57, 58]. ERRα has been found to be under control of myocyte enhancer factor 2 (MEF2) [59], a transcription factor that belongs to the MADS-box superfamily and that activates numerous muscle specific, growth factor-induced and stress-induced genes [60, 61]. Yet, we found a transcript that matches the mRNA of myocyte enhancer factor 2A L homoeolog of *Xenopus laevis* (*mef2a*) to be up-regulated in burst-performant individuals. This transcript has a non-synonymous mutation in the coding part of the MADS-box protein domain (Arg4Lys) which is responsible for DNA recognition and cofactor interaction. Therefore, it is not clear if the *mef2a* transcript of our study negatively regulates *esrra* (and also *ktn1*) expression or if it activates another gene that has yet to be identified. Intriguingly, we found an up-regulation of an inhibitor of cyclin-dependent kinase (CDKI *xic1*) in endurant individuals, while cyclin-dependent kinase (CDK5) has been found to inhibit MEF2 [62].

Several studies have suggested a link between the MEF2 family of transcription factors and calcium-dependent signaling pathways [63, 64]. Calcium signaling is known to be essential for increasing endurance, oxidative capacity, and mitochondrial biogenesis [65, 66]. Likewise, we found an up-regulation in endurant individuals of the calcium/calmodulin-dependent protein kinase (CAMK) 2 A (*camk2a*) along with filamin B (*flnb*), an actin-binding protein (Fig. 4). Interestingly, CAMKs have also been found to activate mitogen-activated protein kinase (MAPK) which mediates early gene expression in response to various cell stimuli. Consistently, *mapk8*, which is up-regulated in endurant individuals, is known to positively regulate the expression of *bnip3*, an apoptosis-inducing protein located in the outer mitochondrial membrane [67]. On the contrary, *bnip3* is negatively controlled by the translation initiation factor 5B (*eif5b)* [68], the latter having an increased expression in burst-performant individuals, along with the ribosomal protein S4 (*rps4x*) and the ribosomal protein S6 kinase α4 (*rps6ka4*). Noticeably, Clarke and colleagues [69] predicted the translation factor *Eif6* to be a key regulator of energy metabolism, affecting mitochondrial respiration efficiency, reactive oxygen species (ROS) production, and exercise performance. Also, *mapk8* and a transcription factor jun-D-like (*jund*) interact with *ddit3* (Fig. 4) which encodes a member of the C/EBP family of transcription factors implicated in adipogenesis, erythropoiesis or promoting apoptosis, and which has two transcript isoforms up-regulated in endurant individuals and one transcript isoform up-regulated in burst-performant individuals.

We found a notable relationship between the calcium signaling pathway and stress-induced genes that are up-regulated in either endurant or burst-performant individuals. This is consistent with previous reports of a link between endoplasmic reticulum (ER) stress, unfolded protein response, and the contractile activity of muscle [70, 71] and suggests a need to further recycle damaged proteins and organelles that are used during muscle activity [72]. For instance, one of those actively used proteins during contraction and relaxation of the muscle is the calcium cycling protein parvalbumin that reduces the free calcium concentration in the sarcoendoplasmic reticulum and cytoplasm [73, 74]. In our study, *ocm4.1,* which codes for a protein that belongs to the paravalbumin family, is significantly up-regulated in burst performant frogs compared to endurant individuals. Similarly, the paravalbumin gene (*pvalb*) was found to be highly expressed in beltfish (*Trichiurus lepturus*), a fish species with high swimming activity [75] and particularly associated with fast contracting muscle fibers [76].

#### Rho-GTPases, ARP2/3 and WAVE complexes, and actin cytoskeleton

Many studies interested in physical performance in general and performance trade-offs in particular, have focused on the physiological aspects of muscle and particularly on fiber type differences [12, 77]. Whereas one major factor contributing to the differences in contractile properties between fiber types is the presence of different myosin heavy chain (MHC) isoforms [78, 79], we found the third most up-regulated transcripts in burst-performant individuals (*LOC108701289)* to be an ortholog of *Xenopus tropicalis speg* gene, which encodes a protein with similarity to members of the myosin light chain kinase family. In addition, we primarily found genes involved in the polymerization and depolymerization of the actin filament to be differentially expressed between endurant and burst-performant individuals. Indeed, the network of actin and actin binding proteins, along with the microtubules and intermediate filaments, constituting the actin cytoskeleton of skeletal striated muscle, is highly dynamic and allows crucial processes like cell migration and division, signal transduction, organelle transport and coordination of muscle contraction [33]. This dynamic system is made possible through the reversible polymerization of globular actin monomers (G-actin) into filaments (F-actin) [80].

Polymerization and depolymerization of the actin filaments is the culmination of a signaling cascade that begins with extracellular stimulation, adhesion interaction (ECM cell-cell interaction) or mechanical stress, which then acts upon guanine-nucleotide-exchange factors (GEFs) and GTPase-activating proteins (GAPs) to control the activation state of the small GTPases Rho, Rac, and Cdc42. After activation, the GTPases bind to a variety of effectors to stimulate downstream signaling pathways [81]. For instance, the non-receptor protein kinase 2 (*ptk2*, up-regulated in burst-performant individuals) can modulate the RhoA regulation pathway, but also activates MAP kinase signaling cascade and mediates activation of the Rho GTPase rac1. Rac1 activates the WAVE regulatory complex that drives Arp2/3 complex-mediated actin polymerization [82]. In our study, *arpc4*, an actin-binding component of the Arp2/3 complex is up-regulated in burst-performant individuals, whereas *abi2*, coding a component of the WAVE complex, is up-regulated in endurant individuals. Interestingly, we found two isoforms of the *nif3l1* gene, one isoform being up-regulated in endurant individuals, the other in burst-performant individuals. This gene may function as a transcriptional corepressor and interacts with *arpc4* and *abi2* in the network involving also *ptk2* and *comp* (Fig. 4). Further examination would be necessary to test the hypothesis that the two isoforms of the *nif3l1* transcript serve as a switch to activate or inhibit proteins of the WAVE and ARP2/3 complex, and thus actin polymerization.

Additionally, we found two up-regulated genes in endurant individuals that are responsible for the actin thin filament length. An F-actin uncapping protein, *lrrc16a,* generates uncapped barbed ends that enhance actin polymerization, as well as a CapZ-interacting protein, *rcsd1,* which induces phosphorylation of CapZIP and regulates the ability of the F-actin-capping protein to remodel actin filament assembly. Antagonistically, the tropomodulin-4 gene (*tmod4*) is up-regulated in burst performant individuals and codes for a type of actin-capping protein that blocks the depolymerization of the actin filaments at the pointed end, thus contributing to the formation of short actin protofilaments. In one network, this gene interacts with a myosin-binding protein C fast-type (*mybpc2*) for which we find two different up-regulated transcripts in burst-performant individuals (Figs. 3, 4). These two transcripts are globally dissimilar, which suggests the presence of two *mybpc2* homoeologs in the allotetraploid *Xenopus allofraseri*. Homoeologs are homologous genes in the same species that started diverging through speciation but were reunified in the same genome by allopolyploidization [83]. The up-regulation of *mybpc2* transcription in burst performant individuals, along with the over-expression of *tmod4*, may lead to the creation and renewal of a short fast-type actin-like filament, necessary for fast and powerful locomotion. Therefore, it seems that there is further depolymerization and polymerization of the actin filaments in respectively burst-performant and endurant individuals, and this may be linked to the need to rapidly recycle and rearrange or to stabilize the actin cytoskeleton.

## Conclusions

Locomotor performance trade-offs have received considerable attention in the literature over the past three decades [11, 13, 84–87], yet the molecular origins of such trade-offs remain unclear. Because locomotor performance is heritable [14–16], substantial effort has been devoted to uncover its molecular basis. However, contradictory results regarding candidate genes suggest that locomotor performance is a complex polygenic trait and that gene expression regulation could be a non-negligible factor. Accordingly, our study reveals numerous transcription factors (DNA and RNA binding), ribosomal proteins, protein kinase, ubiquitin ligase, methyltransferase, and effectors of the signaling cascade that may help explain the trade-off between burst performance and endurance observed in male *Xenopus allofraseri*. Specifically, endurant individuals show an over-expression of protein-coding genes related directly or indirectly to lipid metabolism, mitochondrial activity, ATP production and muscle oxygen supply. Moreover, endurant individuals appear to have increased actin polymerization, whereas burst-performant individuals have increased actin depolymerization along with an up-regulation of two fast-type myosin-binding protein C transcripts. Finally, burst-performant and endurant individuals show several differentially expressed transcripts coding for proteoglycans and extracellular matrix proteins, or proteins involved in intracellular trafficking, apoptosis and the ER stress response. Interestingly, several differentially expressed protein-coding genes are involved in both the calcium signaling and mitogen-activated pathways. How, and if, this relationship could explain the evolution of performance trade-offs remains unclear and would require further investigation.

Previous studies in humans have shown that differences in fiber type can be affected by a stop codon polymorphism (R577X) at *actn3* [88, 89] and this same mutation can alter muscle function in mice [90]. Yet, there are contradictory results regarding the effects of *actn3* polymorphism to explain performance trade-offs in humans [91]. In our study, none of the transcripts corresponding to *actn3* or any member of the α-actin binding protein gene family were differentially expressed. The great variation observed within or between species for muscle fiber type composition can be attributed to the use of alternative splice forms by structural proteins [92]. Indeed, the regulation of, for instance, alternative splicing plays a major role in the production of functional complexity [93] and interestingly, a previous study detected an association between flight performance in dragonflies and alternative splicing in relation to muscle calcium sensitivity [94]. In our study, we found key genes (*akap1* for metabolism, *ddit3* for apoptosis, *ranbp3* for nuclear export, *nif3l1* as a transcriptional corepressor) with transcript isoforms expressed in either burst-performant or endurant individuals. Although our results are biologically meaningful, we want to acknowledge that we were not able to validate these observations by quantitative methods such as RT-QPCR. In addition, RNAseq and qPCR results are known to be closely correlated [95] and the confirmation of these results would rather require testing additional samples. Finally, future studies dedicated to clarify the critical role of alternative splicing and its regulatory mechanism in explaining physical performance trade-offs would be insightful.

## Methods

### Model species

Eight *Xenopus allofraseri* males were caught in the wild (December 2009) in a single pond between Manengoteng (N 04.8090, E 09.8011) and Manjo (N 04.8435, E 09.8218) in Cameroon (Fig. 2). Animals were exported to France with authorization from the Cameroonian Ministry of Forestry and Wildlife (MINFOF) and were housed at the National Museum of Natural History in Paris, France. Frogs were placed in aquaria (60 × 30 × 30 cm) at 24ºC and fed every week with beef heart, earthworms or mosquito larvae ad libitum. All individuals were pit tagged (Nonatec, Rodange, Luxembourg) for permanent identification.

### Physical performance

Performance traits were measured for the eight males at a fixed temperature of 24ºC. Maximal exertion capacity was measured by chasing each individual down a three-meter long circular terrestrial track until exhaustion, as indicated by the lack of a righting response. The floor of the track was covered with moistened cork to improve traction and prevent dehydration. For each individual, we recorded both the total distance covered and time spent moving until exhaustion. Burst performance capacity was quantified by measuring maximal instantaneous swimming speed and acceleration (see additional information about materials and measurement protocol in [8]). From the four physical performance traits, we performed a Principal Component Analysis (PCA) followed by an agglomerative hierarchical clustering using respectively the ade4 and FactoMineR packages of the R software.

### Illumina transcriptome sequencing and de novo assembly

The individuals used for performance measurements were euthanized with a lethal injection of sodium pentobarbital (dosage of 150 mg/kg), a chemical compound acting quickly on the central nervous system, rendering the animal unconscious with little distress (a method validated by the European Commission (https://op.europa.eu/s/olzw). The knee extensor muscles of the right leg were extracted for subsequent RNA sequencing. Tissues were extracted, transferred to labeled tubes containing RNA-later and conserved at − 80ºC until further processing. The protocol for RNA extraction using Trizol, RNA quantification and quality checking can be found in Dhorne-Pollet et al. [96]. PolyA-RNA was isolated and sequencing libraries prepared using ScriptSeq (Illumina). Pooled libraries were 100 paired-end sequenced using an Illumina HiSeq 2500 located at the University of Exeter Sequencing Service facility.

Paired-end sequence reads were pooled together to generate a de novo transcriptome assembly. The raw sequence reads were trimmed and Illumina adapters emoved using Trimmomatic [97] with the following parameters: leading:5 trailing:5 slidingwindow:4:15 minlen:36. Transcriptome assembly was then performed de novo with the program Trinity [98]. We assessed the completeness of our transcriptome assembly by searching for a tetrapod set of 3950 orthologs using BUSCO version 4.0.2 [99]. To obtain assembly quality statistics, paired-end reads were aligned back to the assembly with Bowtie2 [100]. A high-quality transcriptome assembly is expected to have strong representation of the reads input to the assembler and specifically for a trinity transcriptome assembly at least 80% of reads should mapped back to the assembly and exist as proper pairs. Transcript-level abundance was estimated using Kallisto [101], in addition to a normalized measure of transcript expression (TPM).

### Phylogenetic analysis

To evaluate the phylogenetic position of the eight *Xenopus* males, we implemented a phylogenetic analysis with previously published mitogenomes of most known *Xenopus* species downloaded from GenBank [102] (Table S2). We retrieved reads mapping to the mitochondrial genome and performed a reference-based assembly of the complete mitochondrial genome for each sample using Geneious (www.geneious.com) and the *Xenopus allofraseri* mtDNA sequence [98]. We used MAFFT v.7 to compute a multiple alignment of all *Xenopus* mtDNA [103]. From this multiple alignment, we discarded the control region and all positions with too many gaps or misalignments using Gblocks. The phylogeny was then constructed using PhyML v.3.0 [30] including the Shimodaira-Hasegawa (SH) statistic test. For visualization purposes, the phylogenetic tree was designed using Figtree v.1.4.4 [104] (Fig. 2).

### Differential expression analysis

To detect differentially expressed transcripts, we ran edgeR [105], integrated in the R Bioconductor suite [106], using as input the TPM expression values that were cross-sample normalized using the Trimmed Mean of M-values (TMM) method. We chose edgeR rather than DESeq2 as it can detect differentially expressed transcripts between our two conditions even for transcripts that are expressed at low levels, and in cases where there is high variability between the biological replicates, as we observed during data exploration. Differentially expressed transcripts were defined by a log_2_ fold change (log_2_FC) of 2 between burst-performant and endurant groups, and a false discovery rate (FDR) of 0.05. Expression data analysis using Volcano and heatmap plots relied on transcripts identified using the Trinity analysis framework.

### Transcript annotation

We compared the significantly differentially expressed transcript sequences to the combined proteomes of *Xenopus laevis* and *Xenopus tropicalis* extracted from the Uniprot database using BLAST. We manually annotated the transcript sequences lacking detectable protein homologies by comparing them to the nucleotide (nr/nt) database using BLASTN with an *e*-value threshold of 1e^− 5^. Differentially expressed transcripts were analyzed using STRING [107]. The STRING database allows the construction of protein-protein interaction networks, ranging from direct protein-protein interactions to indirect interactions (such as co-expression and text mining). The human database was selected for the network analysis as it contained substantially more information than the one for *Xenopus tropicalis* (the STRING database does not include data for *Xenopus laevis*). Gene symbols for each protein were used to find human orthologues and generate STRING networks using default settings. TSV files with interaction data were then exported and processed using Cytoscape [32] for visual inspection.

## Supplementary Information

**Additional file 1: Table S1.** Up- and down-regulated differentially expressed transcripts (*n* = 103 that match 90 unique protein-coding genes) in the endurant group compared to the burst-performant group based on edgeR method.

**Additional file 2: Table S2.** Species name with their corresponding accession numbers of mitochondrial sequences downloaded from GenBank and used in the phylogenetic analysis.

## Data Availability

The annotation of the 109 differentially expressed transcripts and the STRING TSV interaction file are available on Zenodo (10.5281/zenodo.4028544). Processed and raw data files generated by this transcriptomic analysis have been deposited in NCBI’s Gene Expression Omnibus [108] and are accessible through GEO Series accession number GSE157915 (https://www.ncbi.nlm.nih.gov/geo/query/acc.cgi?acc=GSE157915). The geographic ranges of *Xenopus* species are publicly available at the IUCN red list website (https://www.iucnredlist.org/). Species name with their corresponding accession numbers of mitochondrial sequences downloaded from GenBank and used in the phylogenetic analysis are indicated in Table S2.

## Abbreviations

FDR: False-discovery rate
logFC: Log2 fold-change
mybpc2: Myosin-binding protein C fast-type
PCA: Principal component analysis
FAK: Focal adhesion kinase
FRNK: FAK-related non-kinase
ECM: Extracellular matrix
ROS: Reactive oxygen species
ER: Endoplasmic reticulum
MHC: Myosin heavy chain
GEFs: Guanine-nucleotide-exchange factors
GAPs: GTPase-activating proteins
ATP: Adenosin-triphosphate
RT-qPCR: Reverse transcription quantitative PCR
RNAseq: RNA sequencing
MINFOF: Cameroonian ministry of forestry and wildlife
TPM: Transcripts per million
SH: Shimodaira-hasegawa
TMM: Trimmed mean of M-values
