# Supplementary material for: Transcriptomic analysis of the trade-off between endurance and burst-performance in the frog Xenopus allofraseri
Source: BMC Genomics. 2021 Mar 23;22:204. doi: 10.1186/s12864-021-07517-1 (PMC7986297; doi:10.1186/s12864-021-07517-1)
Supplement: Supplementary file 1 — Additional file 1: Table S1. Up- and down-regulated differentially expressed transcripts (n = 103 that match 90 unique protein-coding genes) in the endurant group compared to the burst-performant group based on edgeR method. [file 12864_2021_7517_MOESM1_ESM.docx]

**Table S1: Up- and down-regulated differentially expressed transcripts (n=103 that match 90 unique protein-coding genes) in the endurant group compared to the burst-performant group based on edgeR method.**

| **Up-regulated** | | | **Down-regulated** | | |
| --- | --- | --- | --- | --- | --- |
| **Gene symbol** | **Log2FC** | **FDR** | **Gene symbol** | **Log2FC** | **FDR** |
| *mfn1* | 10.45 | 0.0159 | *tmod4* | 11.73 | 6.49E-13 |
| *ktn1* | 10.42 | 0.0188 | *baz1b* | 10.49 | 8.08E-13 |
| *LOC108696110* | 10.16 | 0.0017 | *LOC108701289* | 9.82 | 0.0069 |
| *XELAEV_18028904mg* | 10.14 | 0.0210 | *gca.S* | 9.44 | 0.0369 |
| *tp53inp2.S* | 9.09 | 0.0493 | *ptp4a3* | 9.25 | 0.0022 |
| *XELAEV_18004259mg* | 9.05 | 0.0431 | *arpc4* | 8.90 | 0.0002 |
| *srsf7.S* | 9.00 | 8.01E-06 | *arhgdig* | 8.89 | 2.65E-05 |
| *ppox* | 8.82 | 2.21E-06 | *rab12.S* | 8.83 | 0.0369 |
| *XELAEV_18022996mg* | 8.74 | 0.0005 | *mybpc2* | 8.81 | 1.25E-12 |
| *esrra* | 8.73 | 0.0337 | *rps4x* | 8.68 | 2.47E-08 |
| *ddit3* | 8.47 | 8.35E-08 | *bcar3* | 8.54 | 0.0460 |
| *ddit3* | 8.41 | 4.59E-05 | *copz1* | 8.45 | 4.56E-08 |
| *rcsd1* | 8.40 | 0.0004 | *fsd2* | 8.40 | 0.0222 |
| *XELAEV_18006306mg* | 8.38 | 0.0379 | *ascc2* | 8.19 | 0.0114 |
| *ktn1* | 8.27 | 0.0002 | *LOC108703703* | 8.13 | 0.0001 |
| *nfs1.L* | 8.16 | 5.09E-07 | *ptk2.L* | 7.73 | 0.0001 |
| *scoc* | 7.98 | 0.0044 | *rbpms2.L* | 7.49 | 0.0484 |
| *xicl* | 7.92 | 0.0330 | *znf577.S* | 7.47 | 0.0009 |
| *mapk8* | 7.76 | 8.01E-06 | *nectin2* | 7.45 | 4.26E-05 |
| *rmc1* | 7.56 | 7.78E-07 | *nif3l1* | 7.45 | 5.74E-07 |
| *XELAEV_18033460mg* | 7.54 | 0.0053 | *mef2a.L* | 7.40 | 0.0002 |
| *ranbp3.L* | 7.49 | 2.82E-06 | *gtsf1.L* | 7.35 | 0.0435 |
| *rfx5.S* | 7.44 | 0.0002 | *baz2a.L* | 7.26 | 0.0136 |
| *cskmt.L* | 7.42 | 0.0258 | *XELAEV_18015425mg* | 7.19 | 2.47E-08 |
| *nif3l1* | 7.17 | 3.89E-05 | *XELAEV_18006214mg* | 7.14 | 0.0001 |
| *dgat2.L* | 7.13 | 0.0077 | *ranbp3.L* | 7.09 | 8.01E-06 |
| *XELAEV_18027874mg* | 7.12 | 0.0001 | *tubg1.L* | 6.92 | 0.0087 |
| *XELAEV_18044900mg* | 7.12 | 0.0018 | *fbxo3* | 6.91 | 0.0072 |
| *LOC108697220* | 6.98 | 0.0001 | *rps6ka4* | 6.82 | 0.0008 |
| *LOC100158370* | 6.93 | 0.0216 | *XELAEV_18037240mg* | 6.80 | 0.0006 |
| *ctsc* | 6.91 | 0.0330 | *tnk2l.L* | 6.73 | 0.0010 |
| *gpr162.S* | 6.89 | 0.0082 | *cep85* | 6.69 | 0.0092 |
| *LOC108717413* | 6.87 | 0.0010 | *eif5b* | 6.57 | 0.0026 |
| *LOC108701414* | 6.87 | 0.0077 | *XELAEV_18018000mg* | 6.20 | 0.0065 |
| *ddit3* | 6.85 | 0.0320 | *rab12.S* | 6.01 | 0.0005 |
| *fam50a.S* | 6.77 | 0.0072 | *XELAEV_18033899mg* | 5.64 | 0.0419 |
| *rbl1* | 6.77 | 0.0001 | *slc47a2* | 4.89 | 0.0009 |
| *XELAEV_18033636mg* | 6.72 | 0.0067 | *LOC108713478* | 4.52 | 0.0481 |
| **Up-regulated** | | | **Down-regulated** | | |
| **Gene symbol** | **Log2FC** | **FDR** | **Gene symbol** | **Log2FC** | **FDR** |
| *LOC108698702* | 6.67 | 0.0220 | *ctsb* | 4.26 | 0.0208 |
| *XELAEV_18013100mg* | 6.64 | 0.0474 | *ocm4.1* | 4.21 | 0.0062 |
| *narf.L* | 6.62 | 0.0013 | *LOC108709806* | 3.68 | 0.0070 |
| *tspan7* | 6.57 | 0.0127 | *LOC108697262* | 2.65 | 0.0233 |
| *arfgap1.S* | 6.54 | 0.0010 | *rnf19b* | 2.62 | 0.0399 |
| *pnisr* | 6.51 | 0.0415 | *lmo7* | 2.38 | 0.0264 |
| *XELAEV_18030967mg* | 6.47 | 0.0119 |  |  |  |
| *LOC108718747* | 6.47 | 0.0057 |  |  |  |
| *f8a1.L* | 6.35 | 0.0061 |  |  |  |
| *irgq* | 6.28 | 0.0064 |  |  |  |
| *fcp1* | 6.21 | 0.0064 |  |  |  |
| *spib.S* | 5.88 | 2.29E-05 |  |  |  |
| *XELAEV_18006484mg* | 5.82 | 0.0337 |  |  |  |
| *atp5f1b* | 5.41 | 0.0232 |  |  |  |
| *LOC108703539* | 4.89 | 0.0175 |  |  |  |
| *cnksr2* | 4.85 | 0.0010 |  |  |  |
| *LOC108712641* | 4.56 | 0.0113 |  |  |  |
| *nono* | 4.42 | 0.0137 |  |  |  |
| *XELAEV_18012847mg* | 3.67 | 0.0312 |  |  |  |
| *gls2* | 3.21 | 0.0157 |  |  |  |
| *LOC108713633* | 2.12 | 0.0322 |  |  |  |

**Legend:**

Log2FC: Log2 Fold-Change

FDR: False Discovery Rate
