# Supplementary material for: Transcriptomic analysis of the trade-off between endurance and burst-performance in the frog Xenopus allofraseri
Source: BMC Genomics. 2021 Mar 23;22:204. doi: 10.1186/s12864-021-07517-1 (PMC7986297; doi:10.1186/s12864-021-07517-1)
Supplement: Supplementary file 2 — Additional file 2: Table S2. Species name with their corresponding accession numbers of mitochondrial sequences downloaded from GenBank and used in the phylogenetic analysis. [file 12864_2021_7517_MOESM2_ESM.docx]

**Table S2: Species name with their corresponding accession numbers of mitochondrial sequences downloaded from GenBank and used in the phylogenetic analysis.**

| Species name | GenBank accession number/Positions in alignment |
| --- | --- |
| *Xenopus tropicalis* | NC_006839/13-15207 |
| *Xenopus calcaratus* | NC_044865.1/13-15207 |
| *Xenopus mellotropicalis* | NC_044866.1/13-15207 |
| *Xenopus epitropicalis* | NC_044867.1/13-15205 |
| *Xenopus clivii* | NC_044886.1/13-15204 |
| *Xenopus muelleri* | NC_044887.1/13-15185 |
| *Xenopus borealis* | NC_018776/13-15195 |
| *Xenopus fischbergi* | NC_044888.1/13-15161 |
| *Xenopus largeni* | NC_044868.1/13-15204 |
| *Xenopus vestitus* | NC_044881.1/13-15204 |
| *Xenopus lendwensis* | NC_044880.1/13-15203 |
| *Xenopus laevis* | HM991335/13-15200 |
| *Xenopus poweri* | NC_044870.1/13-15203 |
| *Xenopus petersii* | NC_044869.1/13-15203 |
| *Xenopus victorianus* | NC_018775/13-15202 |
| *Xenopus gilli* | NC_044871.1/13-15203 |
| *Xenopus parafraseri* | NC_044872.1/13-15203 |
| *Xenopus itombwensis* | NC_044879.1/13-15203 |
| *Xenopus wittei* | NC_044875.1/13-15203 |
| *Xenopus andrei* | NC_044878.1/13-15203 |
| *Xenopus boumbaensis* | NC_044877.1/13-15203 |
| *Xenopus amieti* | NC_044876.1/13-15203 |
| *Xenopus longipes* | NC_044885.1/13-15204 |
| *Xenopus eysoole* | NC_044884.1/13-15203 |
| *Xenopus ruwenzoriensis* | NC_044882.1/13-15202 |
| *Xenopus kobeli* | NC_044883.1/13-15202 |
| *Xenopus pygmaeus* | NC_044873.1/13-15203 |
| *Xenopus allofraseri* | NC_044874.1/13-15203 |
